# Differential effects of Th1, monocyte/macrophage and Th2 cytokine mixtures on early gene expression for molecules associated with metabolism, signaling and regulation in central nervous system mixed glial cell cultures

**DOI:** 10.1186/1742-2094-6-4

**Published:** 2009-01-21

**Authors:** Robert P Lisak, Joyce A Benjamins, Beverly Bealmear, Liljana Nedelkoska, Diane Studzinski, Ernest Retland, Bin Yao, Susan Land

**Affiliations:** 1 Department of Neurology, 8D University Health Center, Wayne State University School of Medicine, 4201 St Antoine, Detroit, MI, 48210, USA; 2 Department of Immunology and Microbiology, Wayne State University School of Medicine, 540 E Canfield Avenue, Detroit, MI 48201, USA; 3 Department of Biochemistry and Molecular Biology, Wayne State University School of Medicine, 540 E Canfield Avenue, Detroit, MI 48201, USA; 4 Applied Genomics Technology Center, 5107 Biological Sciences, Wayne State University, 5047 Gullen Mall, Detroit MI 48202, USA; 5 Center for Molecular Medicine and Genetics, Wayne State University School of Medicine, 540 E Canfield, Detroit, MI 48201, USA; 6 Department of Surgery, 6C University Health Center, Wayne State University School of Medicine, 4201 St Antoine, Detroit MI, US 48201, USA; 7 Department of Surgery, William Beaumont Hospital, 3601 W Thirteen Mile Rd, Royal Oak MI 48073, USA

## Abstract

**Background:**

Cytokines secreted by immune cells and activated glia play central roles in both the pathogenesis of and protection from damage to the central nervous system (CNS) in multiple sclerosis (MS).

**Methods:**

We have used gene array analysis to identify the initial direct effects of cytokines on CNS glia by comparing changes in early gene expression in CNS glial cultures treated for 6 hours with cytokines typical of those secreted by Th1 and Th2 lymphocytes and monocyte/macrophages (M/M).

**Results:**

In two previous papers, we summarized effects of these cytokines on immune-related molecules, and on neural and glial related proteins, including neurotrophins, growth factors and structural proteins. In this paper, we present the effects of the cytokines on molecules involved in metabolism, signaling and regulatory mechanisms in CNS glia. Many of the changes in gene expression were similar to those seen in ischemic preconditioning and in early inflammatory lesions in experimental autoimmune encephalomyelitis (EAE), related to ion homeostasis, mitochondrial function, neurotransmission, vitamin D metabolism and a variety of transcription factors and signaling pathways. Among the most prominent changes, all three cytokine mixtures markedly downregulated the dopamine D3 receptor, while Th1 and Th2 cytokines downregulated neuropeptide Y receptor 5. An unexpected finding was the large number of changes related to lipid metabolism, including several suggesting a switch from diacylglycerol to phosphatidyl inositol mediated signaling pathways. Using QRT-PCR we validated the results for regulation of genes for iNOS, arginase and P glycoprotein/multi-drug resistance protein 1 (MDR1) seen at 6 hours with microarray.

**Conclusion:**

Each of the three cytokine mixtures differentially regulated gene expression related to metabolism and signaling that may play roles in the pathogenesis of MS, most notably with regard to mitochondrial function and neurotransmitter signaling in glia.

## Background

Genomic analysis has been applied to investigate changes occurring in the central nervous system (CNS) in multiple sclerosis (MS). These include analyses of acute and chronic active lesions, lesions from patients at different stages of MS, and comparisons of normal appearing white matter (NAWM) and normal appearing gray matter (NAGM). Examination of changes in the lesions themselves showed numerous changes in genes related to immune and stress responses, as might be predicted from the pathologic changes in lesions [1-6]. Based on the premise that some of the earliest changes in the pathogenesis of MS lesions would be found in NAWM, where infiltration of immune cells is much less prominent [7,8], Graumann and colleagues [9] analyzed genomic changes in NAWM from patients with secondary progressive MS (SPMS), and found evidence for changes characteristic of neuroprotective mechanisms initially identified in ischemic preconditioning associated with hypoxic insult. Dutta et al [10] examined NAGM and identified reduced expression of nuclear-encoded mitochondrial genes, as well as in genes related to ion homeostasis and neurotransmission. Several of the changes could be localized to neurons but since glia comprise a large proportion of the tissue samples, the relative contribution of neurons and glia to the changes in gene expression could not be quantitated. More recently the same group found upregulation of genes and proteins associated with ciliary neurotrophic factor (CNTF) and signaling pathways in normal cortical gray matter [11]. Subsequently, Mahad, et al [12] found decreased expression of mitochondrial Complex IV cytochrome oxidase subunits COX I and COX IV in type III MS lesions, suggesting that the hypoxia-like damage in this type of lesion may result from mitochondrial dysfunction. These findings suggest that a wide range of metabolic changes occur in both neurons and glia throughout the MS brain, independent of the local presence of systemic inflammatory cells, and that secretory products of immune cells and activated glia may play central roles in the pathogenesis of and protection from both white matter and gray matter damage in MS.

To dissect the underlying molecular changes that might occur in glial cells exposed to secreted products of immune cells, we are utilizing gene array analysis to compare the early effects of mixtures of cytokines typical of Th1 cells, monocyte/macrophages (M/M) or Th2 cells on gene transcription in cultures of mixed CNS glia from rat brain. We have initially focused on changes in gene expression at 6 hours of exposure of CNS glia to cytokines to identify some of the earliest primary responses that might occur in MS brains in response to cytokines, without the confound of changes in gene expression in the inflammatory cells, especially those regulated in the Th1 and Th2 cells. We are currently examining several of the changes in glial cell gene expression by quantitative real time-polymerase chain reaction (QRT-PCR) to analyze the duration of the effects, and find that some changes persist for as long as 5 days [13,14]. In two previous papers, we summarized the effects of these cytokine mixtures on immune-related molecules [15] and on neural and glial related proteins, including neurotrophins, growth factors and structural proteins [16]. Each of the cytokine mixtures induced an unique and complex pattern of changes after 6 hours of incubation. In this third paper, we present the effects of the Th1, M/M and Th2 cytokine mixtures on early gene expression (6 hours) for molecules involved in metabolism, signaling and regulatory mechanisms in CNS glia. A number of the changes found are similar to those found in a gene array analysis of changes in rat spinal cord during the course of myelin basic protein (MBP)-induced experimental autoimmune encephalomyelitis (EAE) [17], including changes in ion homeostasis, mitochondrial function, neurotransmitter-related enzymes, and a variety of signaling pathways. An unexpected finding was the large number of changes in early gene expression related to lipid metabolism.

The culture system we have analyzed is devoid of neurons to enable identification of the responses of the several types of glia to the cytokines in the absence of cross talk with neuronal signaling. For example, although classically thought of as neuron specific, neurotransmitter receptors on glial cells are now known to play prominent roles in glial differentiation [18-22], axonal/neuronal protection [21-26], microglial activation [23] and impulse conduction along myelinated axons [24]. We are initiating studies on enriched neuronal cultures to identify the direct effects of the three cytokine mixtures on early gene expression in neurons for comparison with the changes found in glia, with the goal of identifying those cytokines most supportive of preventing damage and promoting normal axonal function.

## Methods

The methodology has been described in detail in the prior papers [15,16].

### Mixed CNS glial cell cultures

Mixed CNS glial cell cultures were obtained from neonatal rat brain using a modification of the so-called "shake-off" technique [25,26] as we described previously [27]. Following shakeoff of cells from the astroglial bed layer, the time for partial removal of microglia by adherence to plastic was 1 hour prior to plating on poly-lysine coated flasks. Cells were maintained in defined medium containing 2% fetal bovine serum for 6–8 days, then treated with the cytokines. The composition of cultures was examined by indirect immunofluorescence (IF) with antibodies to phenotypic markers for different cells types: glial acidic fibrillary protein (GFAP) for astrocytes [28] (Chemicon, Temecula, CA); galactolipids (GalL) for oligodendrocytes [28,29]; A2B5 for oligodendrocyte precursors [30] (ATCC, Bethesda, MD); ED-1 for microglia [31] (Serotec, Raleigh, NC), Thy1.1 for fibroblasts [32] and in glial cultures some astrocytes [33]; anti-neurofilament heavy chain (NFh) for neurons [34] and anti-factor VIII for endothelial cells (Dako Corporation, Carpinteria, CA).

### Cytokine mixtures

The Th1 cytokine mixture included the rat recombinant cytokines interleukin-2 (IL-2), interferon-γ (IFN-γ) (R&D Systems, Inc, Minneapolis), tumor necrosis factor-α (TNF-α; BD PharMigen, San Diego, CA) and mouse granulocyte-colony stimulating factor (G-CSF; PeproTech, Rocky Hill, NJ).

The M/M cytokine mixture included the rat recombinant cytokines IL-1α and IL-1β, IL-6, IL-12p40 (all from R&D Systems, Inc) and TNF-α. These cytokines would be considered proinflammatory products of M1 macrophages or microglia [35].

The Th2 cytokine mixture included the rat recombinant cytokines IL-4, IL-5, and IL-10 (all from R&D Systems, Inc), mouse G-CSF and purified porcine transforming growth factor-β1 (TGF-β1; R&D Systems, Inc.). In the cognate immune system, in some species, TGF-β1 is considered by some to be the product of so-called Th3 cells. TGF-β1 is also important in the development of another population of T-cells called regulatory T-cells (Treg cells) which are phenotypically characterized as CD4+/CD25 high+/Fox3 [36,37]. These Treg cells may also secrete TGF-β1.

Cytokine mixtures contained 10 ng/ml of each of the constituent cytokines as is typically employed many *in vitro *studies of cytokine biology. For each experiment, four groups of three T75 flasks per group were incubated either with mixtures of Th1, Th2, M/M cytokines or additional medium (control) for 6 hours. Three sets of separate experiments consisting of control, Th1, M/M and Th2 stimulated cultures were performed.

### Cytotoxicity

As reported [15,16], we examined the cytokine-induced effect on cell death in mixed CNS glial cell cultures by incubating cultures from 6 hours to 4 days with the cytokine mixtures. Cell death was determined by uptake of 0.4% trypan blue [38].

### RNA extraction

Cultures were washed and frozen after 6 hours of incubation with cytokine mixtures or additional medium. RNA was extracted employing TRIzol (Gibco BRL, Grand Island, NY) followed by Qiagen RNeasy kits (Qiagen, Valencia, CA). The RNA was quantitated at A_260 nm _and the quality was assessed by at A_260 nm_/A_280 nm_. The 28S/18S ratio was assessed using a Bioanalyzer 2100 (Agilent Technologies, Wilmington, DE), and was > 1.7 for all samples.

### Expression analysis

Biotin-labeled RNA fragments were prepared and hybridized to the Affymetrix rat RG-U34A microarray at 45°C for 16 hours, as previously described [15,16]. Subsequent signal amplification was performed employing biotinylated anti-streptavidin antibody. The RG-U34A chip contains 7,985 genes. The control and three cytokine-incubated cultures from one experiment were analyzed with one gene chip for each sample and three separate experiments using different cultures were analyzed.

### Data analysis

Data were analyzed by comparing the average of the replicates from each of the separate 3 sets of experiments. Affymetrix data were analyzed with dChip v1.2 to correct for background and calculate gene expression values [39]. We analyzed values from 3 separate experiments employing the t-test in GeneSpring comparing Th1, M/M and Th2 with control. Multiple testing analyses that compare all 7,985 genes at different levels of stringency using the Bonferoni and false discovery value (FDV) are statistically most rigorous, but at such high levels of stringency, there were very few changes that reached statistical significance. In order to increase sensitivity and allow identification of potentially important biologic changes, we employed a lower level of stringency [15,16]. In these screening studies at a single time point, we have arbitrarily chosen to represent as probably significant those genes in which the mean expression was > 2 fold (upregulation) or < -2 fold (downregulation) compared to expression in controls (p < 0.2) [15]. We believe this is reasonable given that our experiments consisted of biological replicates that are prone to greater variability than experimental replicates. A similar p value was used in a gene array analysis of MS lesions [2]. The recent literature suggests that a 2-fold cut-off using the Affymetrix platform produces a low false positive rate [40].

### Quantitative real time-polymerase chain reaction (QRT-PCR) expression analysis

Expression of message for iNOS was analyzed by QRT-PCR on an ABI 7500 Fast System, using ABI Taqman rat specific gene expression assays. RNA was extracted as above and reversed transcribed. Relative expression levels were calculated with GAPDH as the internal reference, using the delta-delta Ct method [41]. The values from the treated cultures were compared to those from control. Those ratios were averaged for the three experiments, then expressed as fold changes in the treated cultures relative to control for comparison with the gene array results. Each PCR value represents the average from 2–3 separate experiments.

## Results

### Mixed CNS glial cell cultures

As in our earlier papers, cultures consisted of approximately 35% each of oligodendrocytes and astrocytes and 10% microglia. The remaining cells were glial cell precursors including A2B5 positive oligodendrocyte precursors. Endothelial cells and neurons were not present. Viability was > 98% in all cultures control and cytokine stimulated, at all time points examined (6 hours to 4 days) demonstrating the lack of cytotoxicity under these conditions.

### Overview of cytokine effects on early gene expression

In the preceding papers we first described changes in CNS glia in genes for proteins predominantly associated with the immune system including major histocompatibility molecules, several adhesion and extracellular matrix molecules, cytokines and chemokines and their receptors and complement components [15]. Because of our interests in the effects of cytokines on the production of factors important in oligodendrocyte, axonal and neuronal function, in a second paper we compared the effects of the different cytokine mixtures on expression of genes for neurotrophins, growth factors, related receptors and structural proteins [16]. This third paper summarizes our findings for cytokine-induced changes in glial expression of genes for proteins associated with metabolism, signaling and regulation as well as neurotransmitters and ion channels. As noted, this is a series of screening experiments and therefore Tables 1 and 2 were prepared using the criteria of > 2 fold (increased expression) or < -2 fold (decreased expression) with a p value of < 0.2 for one or two replicates of the gene transcript [15,16]. Unknown genes (ESTs) are not presented.

**Table 1:** Changes in early gene expression: neurotransmitters, ion channels and exchangers, apoptosis, mitochondria and glutathione metabolism

**NEUROTRANSMITTERS AND RECEPTORS**		**Th1**	**M/M**	**Th2**
S50879	acetylcholinesterase T subunit		2.07*	

AF050662	activity and neurotransmitter-induced early gene 10		-3.89*	-5.27**

AF050664	activity and neurotransmitter-induced early gene 12		2.79*	

AF050661	activity and neurotransmitter-induced early gene 9	-2.91*	-4.64**	-3.71*

X57659	adrenergic receptor, alpha 2 c-4	2.53***		

M16406	cholinergic receptor, muscarinic m1		-2.05*	-2.07*

J05231	cholinergic receptor, nicotinic, alpha 5		-2.29***	

L08227	cholinergic receptor, nicotinic, alpha 6	-3.26*		

L31619	cholinergic receptor, nicotinic, alpha 7		-2.14**	-2.29**

L31622	cholinergic receptor, nicotinic, beta 2		-2.33**	

M35077	dopaminergic receptor A1	-12.11***		-14.19***

A17753	dopaminergic receptor D3	-10.31***	-5.21*	-8.28**

L08493	GABA-A receptor, alpha 4		-2.0*	

S56679	glutamate receptor, AMPA-selective A			-3.31***

U08255	glutamate receptor, ionotropic, delta 1	2.68****		

AF027331	glutamate receptor, ionotropic, kainate 5		2.15*	

D13211	glutamate receptor, ionotropic, NMDA 2A		-3.02**	-3.50***

X96790	glutamate receptor, metabotropic 7b.	-9.46****	-3.02**	-3.50***

D16817	glutamate receptor. metabotropic 7		-2.19*	

U28504	glutamate transporter, vesicular, family 17	-3.29*		

X55246	glycine receptor, alpha 1			-3.05****

D00833	glycine receptor, alpha 1 subunit	-2.21*	-7.08****	

X57281	glycine receptor, alpha 2 subunit	-2.36***		

L13600	glycine transporter 1	2.86***		2.12***

U66274	neuropeptide Y receptor 5	-18.76**	-8.72*	

X56306	preprotachykinin A (substance P precursor)			-7.17**

L46874	proton-driven peptide transporter.	-2.10**	-3.50***	

X90561	purinergic receplor P2X, ligand-gated 3			-5.56*

X80477	purinergic receptor P2X, ligand-gated 1	-3.23*		-3.0***

AF020758	purinergic receptor P2X, ligand-gated 2		-3.86*	-3.81*

X95882	purinergic receptor P2X, ligand-gated 7		-2.10*	

U56839	purinergic receptor P2Y, G-protein coupled 2		3.51***	2.41***

Y311433	purinergic receptor P2Y, G-protein coupled 4			-2.53*

X66842	serotonergic receptor 2B	-2.09***		

U20907	serotonergic receptor 4			-6.05****

**ION CHANNELS**				

M99222	Ca++ channel, A/P type, alpha 1, splice variant	-3.75*		

M57682	Ca++ channel, voltage-gated, L type, alpha 1D	-4.62***	-3.39*	-7.36***

D26111	Cl- channel (ClC-K2L and ClC-K2S), splice variant	-3.13*	-2.02*	

X62894	Cl- channel, voltage-gated, 1 (skeletal muscle)		-2.80**	-2.59*

X78461	K+ inwardly-rectifying channel, J12 (kir 3.3/IRK3)		2.05*	

U69884	K+ channel, small conductance Ca++-activated	-3.17*		-2.55*

L35771	K+ channel, inwardly-rectifying, J5			-3.56***

L77929	K+ channel, inwardly-rectifying, J9			-2.49*

U40603	K+ channel, large conductance Ca++activated M alpha1	-3.50*		-3.61*

AB010963	K+ channel, large conductance Ca++-activated M beta1			-2.20***

D10709	K+ channel, lsk1 (epithelial), E1			2.50***

AF031384	K+ channel, voltage-gated, K3		-2.45*	-2.36**

AF087453	K+ channel, voltage-gated, KQT-like 2		-3.39*	

AF087454	K+ channel, voltage-gated, Q3		-2.56*	-2.18*

Y17606	K+ channel, voltage-gated, S1	-4.17**	-3.80*	-4.68*

J04731	K+ channel, voltage-gated, shaker related 2	-7.17*		

X16003	K+ channel, voltage-gated, shaker related 2.		-2.44*	

U72410	K+ inwardly-rectifying channel, J3 (GIRK1)			2.75***

M22253	Na+ channel, voltage-gated, 1 alpha	-9.23***	-6.92**	

M91808	Na+ channel, voltage-gated, 1 beta			-2.46****

AF000368	Na+ channel, voltage-gated, 9 alpha	-3.36*		

AA891751	Na+ channel, voltage-gated, 3 alpha (CIN3)			-2.10**

Y00766	Na+ channel, voltage-gated. 3 alpha (CIN3)			-2.76**

**ATPase ION EXCHANGERS**				

M99223	Ca++ ATPase, cardiac, fast twitch 1	-3.80**	-2.82*	-2.70**

AA800212	Ca++ ATPase, cardiac, slow twitch 2	-2.06*		-2.35*

Al172499	Ca++ ATPase, plasma membrane 1		3.0***	

X76452	Ca++ ATPase, plasma membrane 4			-2.06***

AA956437	ER ATPase, peroxisome biogenesis factor 1	-2.35*		

U94911	H+/K+ ATPase, nongastric, alpha 2a		-2.30***	

M90398	H+/K+ ATPase, nongastric, alpha 2a		-2.73*	

U15176	Na+/K+ ATPase, alpha 4	-3.15*		-2.86*

**APOPTOSIS**				

H31839	Bcl X	2.35**		

AF025671	caspase 2		-2.93****	-2.63***

AF072124	caspase 7	3.71***	3.17**	

M33605	cytolysin	-2.06***		-2.22***

Al639313	huntingtin associated protein interactive protein	-2.40***	-2.34*	

Al176462	programmed cell death 2		-2.47****	

**MITOCHONDRIA**				

AJ007488	16s ribosomal RNA, mitochondrial	-4.1****		-2.81**

X72758	COX VIa2, Complex IV	-3.16*		

M20183	COX VIc1, Complex IV	-3.76**		

M10140	creatine kinase (muscle)		-2.02*	-2.01*

Al044488	ferredoxin 1	-2.27*		-2.61*

D26393	hexokinase II	2.75*		

X87884	mitochondrial capsule selenoprotein.			-2.88*

AA799479	NADH dehydrogenase (ubiquinone), Complex I		-2.43***	

AA891651	NADH dehydrogenase (ubiquinone), Complex I	-2.20*	-2.61***	

AI75973	NADH dehydrogenase (ubiquinone), Complex I			-2.61*

X59736	sarcomeric mitochondrial creatine kinase.		-5.18*	

Y00497	superoxide dismutase 2	3.79***	5.82****	2.00**

X68041	superoxide dismutase 3	-2.96**		

X59793	ubiquitous mitochondrial creatine kinase		-2.61**	

A04674	uncoupling protein 1, proton carrier	-2.19***		

**GLUTATHIONE-RELATED**				

U73174	glutathione reductase	2.07*	2.74**	

AI138143	glutathione S-transferase, theta 2	-2.04**		

S72506	glutathione S-transferase, Yc2 subunit		-5.0**	-3.01*

AI235747	glutathione-S-transferase, alpha (Ya)		-2.36*	-2.22***

M81855	P-glycoprotein, multi-drug resistance 1	5.45**	5.10***	

Values represent averages of fold changes from three separate experiments for each cytokine mixture compared to control. The accession numbers are from Genbank.

****p < 0.01; ***p < 0.05; **p < 0.10; *p < 0.20.

**Table 2:** Changes in early gene expression: gene regulation, signaling, cytoplasmic transport and metabolism

**TRANSCRIPTION FACTORS**				
U04860	aryl hydrocarbon receptor	-2.48****	-2.66**	

X14788	CREB	2.46*		2.02*

Hi1677	eIF5 (elongation initiation factor 5)		2.47**	

Al03194	eIF5 (elongation initiation factor 5)		2,06***	

Al638955	fox-1 homolog	4.83**		4.08****

L13261	hepatic nuclear factor 3 (forkhead homolog 1)		2.09***	

L09647	hepatic nuclear factor 3 beta	-2.23**	-2.29*	-2.35**

AB01774	hepatic nuclear factor 3 gamma			-2.21*

X57133	hepatic nuclear factor 4 alpha	-2.61**	-4.80****	-3.46****

X57133	hepatic nuclear factor 4 alpha	-6.63***	-3.69***	-2.92**

D10554	hepatic nuclear factor 4 alpha			-2.38**

AA891041	junB	4.49***		2.48**

X54686	junB	2.29****	2.83****	2.14****

L26267	NFkappa B, p105 subunit	3.49**	3.97***	

L23862	POU domain		-2.47**	

**NUCLEAR RECEPTORS**				

U40064	PPAR delta	2.31**	2.06**	

AB011365	PPAR gamma	-5.41****	-4.52****	-2.09*

**SIGNALING**				

L26986	adenylyl cyclase, type 8		-4.11*	

U73503	calmodulin kinase II, gamma E	-2.26*		

U09583	fyn-related kinase (src homology)			

AB007688	homer	4.03*	4.03****	

AA900503	jagged 1	2.15*	2.35*	

L38483	jagged 1		2.38***	

U13396	Janus kinase 2 (JAK 2)	5.20***	4.97***	

U13396	Janus kinase 2 (JAK 2)	2.34***		

AJ000557	Janus kinase 2 (JAK 2)		3.36***	

L14951	lyn protein non-receptor kinase	2.45**	2.00*	

L14782	lyn protein non-receptor kinase	2.17***		

AA946094	lyn protein non-receptor kinase	2.14**		

X96488	MAP kinase 12	-2.07*		

M95437	phosphodiesterase 1B		-2.36**	

AL235758	protein kinase A, 2, regulatory subunit		-2.15*	

M18330	protein kinase C, delta	2.11**		

U69109	protein tyrosine kinase 2B	4.59**	3.17**	

AF063890	protein tyrosine kinase 2B	3.30***		

Al113289	protein tyrosine phosphatase, non-receptor	2.31***	2.45***	

AF05398	ras GTPase activating protein	-2.04**		

U69702	receptor serine threonine kinase	-2.50*	-2.56*	

U69702	receptor serine threonine kinase		-2.56*	

AF097887	Rho family GTPase		-2.07***	

AA892553	STAT 1	10.30***		

X91810	STAT 3	2.49****	2.42****	

U24175	STAT 5a	3.13***	4.41***	

L27112	stress activated protein kinase alpha II			2.85***

**CYTOPLASMIC TRANSPORT AND DEGRADATION OF PROTEINS**				

AF077354	heat shock 70 kDa protein 4		2.24**	

AA799492	proteasome (prosome, macropain) alpha 6	9.69****		

AA891383	proteasome (prosome, macropain) alpha 6	3.29***		

D45249	proteasome activating subunit alpha	2.12***		

D45250	proteasome activating subunit beta	3.68***		

D21799	proteasome subunit RC7-1	2.12***		

D10757	proteasome subunit R-RING	6.76****		

D10757	proteasome subunit R-RING	6.45**		

AJ224441	proteasome subunit R-RING	-3.62**	-3.78***	

H31236	similar to ubiquitin-conjugating enzyme E2D 2			-2.50*

S73007	synuclein alpha (SYN 1)	2.87**		

S73008	synuclein beta (SYN 2)	-3.82**	2.58**	

AA874859	ubiquitin ligase (NEDD 4)	-2.25*	-3.19***	

L38482	ubiquitin-conjugating enzyme		2.01**	

U56407	ubiquitin-conjugating enzyme E2D 2	-4.18***	-6.38***	-4.76***

AA685152	ubiquitin-like protein (NEDD 6)	2.71***	2.90***	

**LIPID SYNTHESIS**				

S78687	3-OH-3-methylglutaryl CoA reductase	-2.34**	-2.34**	

J02749	acetyl CoA acyl transferase		-2.34*	-2.29*

J03808	acetyl CoA carboxylase	-2.40*		

AB010428	cytosolic acyl CoA thioesterase 1	3.91*		

M95591	farnesyl phosphate farnesyl transferase		-2.11***	

Al004900	fatty acid CoA ligase, long chain 2	2.47****		

AA925424	fatty acid CoA ligase, long chain 4	-4.83***	-4.27****	

M33648	mitochondrial 3-OH-3-methylglutaryl CoA synthase			-2.72**

Y09333	mitochondrial acyl CoA thioesterase	2.68**		

D00512	mitochondrial fatty acid acetyl CoA thiolase	-2.39*		

AA800303	phospholipid scramblase	3.16*		

U07683	UDP-galactose ceramide galactosyl transferase	-3.14*		

AF047707	UDP-glucose:ceramide glycosyltransferase	2.57***	2.61***	

**LIPID SIGNALING**				

AB00999	CDP-diacylglycerol synthase	9.91****	6.45****	

AB00999	CDP-diacylglycerol synthase	2.52***		6.40****

AA818983	diacylglycerol kinase beta	-4.21**		

U10303	EDG, endothelial sphingolipid GPCR		-6.17****	-2.65*

AA859981	myo-inositol monophosphatase		-2.01***	

D88666	phosphatidyl serine specific phospholipase A		3.08**	

D00036	phospholipase A2, group 1B		-4.15*	

U03763	phospholipase A2, group 5		-4.17**	

L14322	phospholipase C beta 1	-2.37**		

**STEROID AND VITAMIN D RELATED**				

U89280	17-beta hydroxy sterol reductase	-2.81**		-3.53****

X97754	17-beta hydroxy sterol reductase, type 1	-4.53***		

L04619	25-OH vitamin D3 24-hydroxylase (Cyp24a1)		-5.20*	-4.42*

M95058	steroid 5-alpha reductase 2	-2.04**		

M13646	testosterone 6-beta hydroxylase		-6.91***	-2.03*

Y07534	vitamin D3 25-hydroxylase (Cyp27a1)	2.49*		

**MISCELLANEOUS**				

X02361	alpha-fetoprotein	-2.50*	-2.56*	-2.49*

AA998229	alpha-fetoprotein			-2.22**

M90065	angiotensin receptor, type 1b			-5.02****

U01908	angiotensin receptor, type 2	-3.82**		

J02720	arginase 1			4.82***

X03369	beta tubulin 2b	-4.09*	-4.90*	-11.96***

U60758	carbonic anhydrase II	2.46*		2.10*

Al043968	caveolin 3		-2.44*	

AA817854	ceruloplasmin		2.56*	

L33869	ceruloplasmin		2.04*	

U49049	chapsyn-110	-4.05*	-4.77**	-29.4****

U09538	fyn-related kinase (src homology)		-2.49**	

J03624	galanin	5.03**	3.22***	

S57565	histamine H2 receptor	-2.62**		-2.17**

M11566	kallikrein S3	-2.34**	-2.48**	

M27217	kallikrein-related (rGK-8)		-2.88**	

AB005900	low density lipoprotein receptor 1	4.06**	2.18**	5.84**

AI07531	low density lipoprotein receptor 1	3.19****		5.40***

AI030685	nestin	-2.77*		

U03699	nitric oxide synthase 2 (iNOS)	14.69***	14.61***	

AA892953	similar to carbonic anhydrase		2.19*	

U09361	VIP receptor 2	-3.14*		

Values represent averages of fold changes from three separate experiments for each cytokine mixture compared to control. The accession numbers are from Genbank.

****p < 0.01; ***p < 0.05; **p < 0.10; *p < 0.20.

### Neurotransmitters and receptors

All three cytokine mixtures had regulatory effects on message levels for a wide range of message levels for neurotransmitters and their receptors as well as on transporters involved with transmitters including glutamate, adrenergic, cholinergic, glycine, serotonergic, dopaminergic and purinergic systems (Table 1). The only adrenergic receptor affected was alpha 2 c-4, upregulated 2.5 fold (p < 0.05) by Th1 cytokines. Among cholinergic receptors, the largest change was for nicotinic cholinergic receptor alpha5, downregulated -2.3 fold (p < 0.05) by Th1. Dopaminergic receptors A3 and D1 were markedly downregulated -8 to -14 fold by Th1 and Th2 cytokines. Among several changes in glutamate receptors, Th1 upregulated ionotropic glutamate receptor delta 1 by 2.7 fold (p < 0.01), but markedly downregulated metabotropic glutamate receptor 7b by -9.5 fold (p < 0.01). Neuropeptide Y receptor 5 was downregulated by both Th1 and MM cytokine, -18 fold (p < 0.10) and -8 fold (p < 0.20), respectively, while the substance P precursor preprotachykinin A was downregulated -7 fold (p < 0.10) by Th2. For purinergic receptors, the most robust changes were -3 fold (p < 0.05) downregulation of P2X1by Th2, and upregulation of P2Y2 by MM and Th2, 3.5 fold and 2.4 fold, respectively, both p < 0.05.

### Ion channels

Th1, M/M and Th2 cytokines had primarily downregulatory effects on expression of a very large number of genes for proteins that are components of ion channels including Na, K, Ca and Cl channels, both voltage-gated and non-voltage gated (Table 1). For example, Th1 and Th2 downregulated the voltage-gated alpha 1D L type Ca++ channel by -4 and -7 fold respectively, both p < 0.05. A large number of K+ channels were downregulated by Th2 cytokines, with fewer downregulated by Th1 or MM cytokines. The voltage-gated 1 alpha sodium channel was robustly downregulated by Th1 and MM cytokines, -9 fold (p < 0.05) and -7 fold (p < 0.10) respectively, while Th2 cytokines uniquely downregulated the 1 beta isoform, -2.5 fold, p < 0.01.

### ATPase ion exchangers

In addition to the effects on ion channels shown in Table 1, there were effects on several ATPase ion exchangers. With the exception of upregulation of Ca++ATPase (plasma membrane 1) by M/M cytokines, several ATPase ion exchangers were downregulated by each of the cytokine mixtures (Table 1).

### Apoptosis

The cytokine mixtures induced up and down regulation of several genes for proteins involved in control of apoptosis (Table 1) including caspase 2, downregulated -3 fold, p < 0.05 by both MM and Th2 cytokines, and caspase 7, downregulated -3 fold by Th1 and MM cytokines.

### Mitochondria and related proteins

There were cytokine mixture-induced changes in expression in genes of some mitochondrial proteins which are listed in Table 1, including a 4–6 fold (p < 0.05) upregulation of super oxide dismutase 2 (SOD2) by Th1 and MM, and an apparent -4 fold (p < 0.01) decrease for 16S mitochondrial ribosomal RNA.

### Glutathione-related

The genes for several proteins involved in glutathione metabolism and secretion were regulated by the different types of mixtures of cytokines (Table 1). Subunits of glutathione S-transferase were generally downregulated, while both Th1 and MM upregulated P-glycoprotein(multi-drug resistance protein) by 5 fold, p < 0.10 and p < 0.05, respectively.

### Transcription factors

Th1 cytokines markedly upregulated junB, CREB and the p105 subunit of NF κB. Both Th1 and M/M cytokines altered expression of genes for several other transcription factors, while the Th2 cytokines had minimal effects (Table 2). All three cytokines markedly upregulated message levels of junB (2–3 fold, p < 0.01) and downregulated hepatic nuclear factor alpha (-3 to -7 fold, p < 0.05.). Both Th1 and M/M cytokines upregulated the expression of the gene for NF kappa B p105 subunit and downregulated the aryl hydrocarbon receptor (Table 2).

### Nuclear receptors

Th1 and M/M cytokines upregulated the gene for peroxisome proliferator activator receptor δ (PPAR δ) by 2 fold (p < 0.10), and downregulated the gene for PPAR γ by -5 fold (p < 0.01). Th2 cytokines downregulated the gene for PPAR γ but had no effect on PPAR δ (Table 2).

### Signaling

The cytokine mixtures had effects on the expression of genes for many signal transduction molecules, some of which are presented in Table 2. Among these are STAT1, 3 and 5, JAK2, homer and protein kinase 2 B. Of interest, only one gene in this category was affected by Th2 cytokines, stress activated protein kinase alpha 2, upregulated 3 fold (p < 0.05).

### Cytoplasmic transport and degradation of proteins

Th1, M/M and Th2 cytokines had effects on the genes for several proteins involved in synthesis, degradation and intracellular transport of proteins including synucleins, proteasome subunits, ubiquitin conjugating enzymes and heat shock protein (HSP) 70 kDa (Table 2). For example, proteasome (macropain) alpha 6 was upregulated 10 fold (p < -.01) by Th1 cytokines, while the ubiquitin-conjugating enzyme E2D 2 was downregulated -4 to -6 fold (p < 0.05) by all three cytokine mixtures.

### Lipid synthesis and signaling

As noted in Table 2, the genes for several proteins involved in lipid metabolism and signaling were regulated by the different cytokine mixtures. For example, fatty acid CoA ligase long chain 4 was downregulated -4 to -5 fold (p < 0.05 fold) by Th1 and Mm cytokines, while UDP-glucose ceramide glycosyl transferase was upregulated 3 fold (p < 0.05). With regard to lipid signaling, CDP diacylglycerol synthase was markedly upregulated 6–10 fold (p < 0.01) by all three cytokines, while EDG (endothelial sphingolipid GPCR) was downregulated -6 fold (p < 0.01) by MM cytokines.

### Steroid and vitamin D related

Specific genes regulating proteins involved in sterol and vitamin D metabolism were also regulated by the cytokine mixtures (Table 2). Thus, 17-beta hydroxyl sterol reductase was downregulated -4 to -5 fold (p < 0.05) by Th1 and Th2 cytokines, and testosterone 6-beta hydroxylase was downregulated -7 fold (p < 0.05) by MM cytokines.

### Miscellaneous proteins

There were a large number of genes for proteins not included in the above categories that are potentially of importance in understanding the pathogenesis of MS, as well as protective and reparative mechanisms. These are listed in Table 2. Of note, Th1 and MM cytokines upegulated nitric oxide synthase 2 (iNOS) by 15 fold (p < 0.05), while Th2 cytokines markedly downregulated genes for angiotensin receptor type 1b (-5 fold), beta tubulin 2b (-12 fold), and chapsyn-110 (-29 fold), all p < 0.05, but upregulated arginase and low density lipoprotein receptor 1 by 5 fold (p < 0.05).

### QRT-PCR

We validated expression changes in three genes by QRT-PCR: iNOS, the enzyme that synthesizes NO from arginine; arginase, the enzyme that breaks down arginine, thus limiting production of NO; and P-glycoprotein (multi-drug resistance 1), an ABC transporter involved in regulation of glutathione levels. As noted in Table 3, we were able to confirm striking upregulation of the gene for iNOS by Th1 and M/M at 6 hours employing QRT-PCR. Although no effect on the gene for iNOS expression was observed at 6 hours in response to Th2 cytokines on microarray, we detected modest downregulation employing QRT-PCR. For arginase, we confirmed upregulation by Th2, with no change induced by Th1; however, in contrast to the array results, PCR indicated some upregulation of arginase by MM at 6 hours, rather than no change. For P glycoprotein, PCR showed upregulation by Th1 (in two of three analyses) and MM, as on the gene array, but also indicated a modest increase with Th2 rather than no change. The results for these three genes show relatively good agreement, and indicate that the arrays are not giving false positive results, but in some instances may give false negative results, suggesting that PCR may be more sensitive than the gene array.

**Table 3:** QRT-PCR validation of gene array results for cytokine-induced changes in gene expression at 6 hours

**Gene**	**Cytokine**	**Array**	**PCR**
**iNOS**			
	Th1	14.7	35.9 +/- 18.8
	M/M	14.6	371 +/- 146
	Th2	NC	-3.7 +/- 0.9
			
**arginase**			
	Th1	NC	1.05 +/- 0.28
	M/M	NC	5.37 +/- 2.03
	Th2	4.82	20.9 +/- 5.16
			
**P glycoprotein**			
	Th1	5.4	2.51 +/- 2.54
	M/M	5.1	3.77 +/- 1.84
	Th2	NC	1.99 +/- 0.28

RNA extracts from 2 experiments for iNOS and 3 experiments for arginase and P glycoprotein were analyzed by QRT-PCR, as described in Methods. Values are averages +/- range. In agreement with the gene array results, Th1 and M/M cytokines markedly upregulated expression of iNOS in the mixed glial cultures; Th2 cytokines upregulated arginase; and Th1 and M/M cytokines upregulated P glycoprotein. Differences from the gene array were a decrease in iNOS by Th2, an increase in arginase by M/M and an increase in P glycoprotein by Th2 cytokines.

Values for iNOS, +/- range; values for arginase and P glycoprotein, +/- S.D.

## Discussion

In our two preceding papers, we showed marked differential early effects of Th1 cytokines, M/M cytokines and Th2 cytokines on glial expression of a variety of genes, including those for immune-related molecules [15] and for neurotrophins, growth factors and structural proteins [16]. In addition, following the 6 hours of cytokine exposure used in these studies, we saw changes in expression of a large number of genes involved in signaling, regulation and metabolism. Some of these changes might be predicted from known effects of cytokines in vitro and in EAE or MS tissue, while other changes were unexpected. In contrast to the in vivo studies, our examination of an early 6 hour time point provides information about what might be some of the initial responses of glia *per se *to these cytokines.

### Neurotransmitters and receptors

Glial cells have been reported to express different neurotransmitters and receptors as well as transporters for these transmitters [23,42-45]. With increasing evidence that both oligodendrocyte and neuronal/axonal damage may be caused by glutamate induced toxicity [46-50], and that other glutamate receptors may be protective [51], the effects of cytokine mixtures on different GluR may influence and modify the effects of glutamate. AMPA, kainate and NMDA receptors may be important in oligodendrocyte toxicity in MS and EAE [52-57] whereas upregulation of metabotropic GluRs (mGluRs) may provide protection [58]. The effects of upregulation of AMPA, NMDA and kainate GluR on neuronal death are well recognized [59]. It is of interest that Th1 and M/M upregulated GluRs associated with cell toxicity whereas all three cytokine mixtures markedly downregulated metabotropic mGluR7b. The group III mGluRs, including mGluR7, inhibit production of RANTES induced in astroglia by TNF-α or IFN-γ [60]. We previously reported upregulation of the gene for RANTES by Th1 and M/M cytokines [15].

The role of other transmitters in glial cell function is not as well understood. In addition to receptors for well established neurotransmitters and classically described ion channels (Ca++, K+ and Na+), we detected effects on genes for the purinergic P2X receptors, some of which serve as ligand gated ion channels [61], and the P2Y receptors which act as G protein coupled receptors when ligated by extracellularly released nucleotides, as occurs in inflammation and other stressful conditions within the CNS. The purinergic receptors modify the response of astrocytes to cytokines such as IL-1β and TNF-α and modify astrocyte functions including Ca++ influx [62-64], as well as modulating transport of other ions [65]. The P2X7 receptor is found in resting and activated microglia in epileptic brain and several other neurologic diseases [66], and plays a role in microglial proliferation [61] and migration [23]. The P2X7 receptor is expressed in reactive astrocytes and microglia/macrophages in MS lesions [62], and is transiently upregulated by the M/M cytokine IL-1β in cultured fetal human astrocytes, resulting in increased iNOS activity [64]. We noted modest downregulation of the gene for the P2X7 receptor with M/M at 6 hours in our mixed glial cultures, suggesting that the mixture of cytokines or the presence of other glial cell types may modulate the glial responses to IL-1β, or that upregulation seen *in vivo *and *in vitro *in other studies may be a secondary response occurring at later time points.

The different cytokine mixtures had variable effects on a large number of receptors for several other transmitters including dopaminergic, serotonergic, cholinergic, adrenergic and melanocortin as well as the previously discussed purinergic receptors. The downregulation of the genes for the D1 and D3 dopamine receptors by the cytokine mixtures was especially striking. All three types of glia are known to express dopamine receptors, and D3 dopamine receptors may play roles in oligodendroglial differentiation and myelination [67], as well as protection of oligodendrocytes against glutamate oxidative stress and oxygen glucose deprivation [68]. Binding of neurotransmitters to their receptors on microglial cells seem to be important in microglial function [23].

It is also of interest that several of the neuronal nicotinic acetylcholine receptors are involved in downregulation of proinflammatory immune reactions in the systemic immune system, in particular the nicotinic α7 receptor [69,70], which we found downregulated by both M/M and Th2 cytokine mixtures. Further, attenuation of cholinergic signaling with the acetylcholinesterase inhibitor physostigmine results in inhibition of CNS inflammation and development of EAE [71]. Conversely, we found that M/M cytokines upregulated the gene for the acetylcholinesterase T subunit, which could lead to increased acetylcholinesterase and a decreased "protective" cholinergic response.

Expression of genes for several transporters for glutamate and glycine were also observed along with changes in the genes for the R5 receptor for neuropeptide Y receptor 5 and preprotachykinin A (precursor of substance P). The role of such receptors and transporters in glial cells is not clear. Of interest, changes were found in activity of neurotransmitter-induced early genes 9, 10 and 12. It is not known if these cytokine mixtures have a similar effect on the same genes and their proteins in various subpopulations of neurons. If they do, this would have major implications on neuronal and axonal dysfunction in MS and other diseases characterized by CNS inflammation and/or microglial activation [72], as well as symptoms in patients with MS such as depression, memory loss, abnormalities in other cognitive functions, fatigue and pain.

### Ion channels

We observed cytokine induced changes in gene expression for many ion channels including Na+, K+ and Ca++ channels. It is well established that glial cells have a wide variety of ion channels which are important in glial cell function [21,73-77] and that expression of some of these have been reported to be affected by cytokines in glial cells and neurons, as well as other types of cells [21,78-81]. Cytokine effects on ion channels and ion exchangers clearly are important in axonal and neuronal function and viability as well as likely contributing to symptomatology in patients with MS [82-86]. Changes in genes for ion channels have been reported in the CNS in MS and EAE [2,87].

There have been reports of inflammatory mediators such as inducible nitric oxide synthase (iNOS) inducing upregulation of certain Na channels in neurons [84,88]. We observed significant effects on gene expression for a wide variety of ion channels in glial cells at 6 hours of incubation suggesting a direct effect of cytokines on expression of genes for some or all of these channels in glia. Changes in the distribution of ion channels could contribute to glial cell dysfunction. If similar changes were induced directly in neurons/axons, these changes could contribute to plasticity as well as to axonal and neuronal cell death. While such changes in neuronal ion channels and failure and reversal of ion exchangers, particularly Ca++ exchangers [89,90], could result from failure of mitochondrial energy metabolism [10,91], our results also raise the possibility of axonal dysfunction and ultimately death by direct effect of cytokines on expression of genes for ion channels and ATPase ion exchangers (see below). The cytokine mixtures also likely regulate ion channels on inflammatory cells such as lymphocytes, and ion channels are known to affect lymphocyte function [92].

### ATPase ion exchangers

Th1 downregulated the genes for Na+/K+ ATPase, α4 polypeptide and Ca++ transporting ATPase. M/M upregulated the genes for Ca++ transporting ATPase and Ca++ ATPase, plasma membrane 1. Th2 likewise downregulated the genes for several ATPase ion transporters including Na^+^/K^+ ^ATPase α4 polypeptide, Ca++ transporting ATPase, Ca++-pumping ATPase isoform 4, H+/K+-ATPase α2 gene, alternatively spliced and H^+^/K^+ ^ATPase, nongastric, α polypeptide, nongastric; and the Na^+^/H^+ ^ion exchanger (Table 1).

### Apoptosis

The possible role of oligodendrocyte cell death through apoptosis via caspases [93] or via other pathways to cell death [94] in MS lesions continues to be controversial, and it is likely that apoptosis as a mediator of oligodendrocyte death varies in different lesions [95]. Neuronal cell death by what appears to be apoptosis is also seen [96]. Up and downregulation of expression of various genes for proteins involved in apoptosis including caspases and Bcl X were noted. Th1 and M/M cytokines both induced upregulation of the genes for caspase 7, a downstream effector caspase involved in caspase-dependent apoptosis [97], and Bcl X, a protein which inhibits apoptosis [98,99]. M/M cytokines downregulated the gene for caspase 2, a caspase implicated in oligodendrocyte cell death via the p75 receptor [93]. The gene for cytolysin (a constituent of lymphocyte toxic granules) was downregulated by *both *Th1 and Th2 cytokines, and Th2 *and *M/M cytokine mixtures both downregulate the gene for the protein programmed cell death 2.

Mutations in the gene for the protein huntingtin (Htt) result in Huntingon's chorea. Htt interacts with several proteins. One of these proteins is called htt-interacting protein (HIP-1). When HIP1 is bound to normal Htt, it inhibits apoptosis in certain neurons and Htt seems to be involved in endocytosis as well [100-103]. In addition abnormal huntingtin interferes with normal ubiquitin-proteosome function and one could readily postulate that downregulation of proteins such as HIP-1 that interact with htt could also lead to abnormal protein aggregation such is seen in many degenerative diseases including Huntington's disease, where it is the htt that is qualitatively abnormal [104]. There was downregulation of the gene for HIP-1 by Th1 and M/M cytokines.

Changes in expression of mitochondrial protein genes, including genes associated with some apoptotic pathways, were noted (see below).

### Mitochondria and related proteins

Changes in mitochondrial related genes have been noted in MS cortical gray matter in patients with long-standing chronic MS [10], and failure in mitochondrial associated energy metabolism may be important in axonal and neuronal degeneration and cell death [89,91]. Most of the detected changes were reduced expression of genes, particularly components of complex I, III and IV. Decreased expression of COX subunits I and IV (Complex IV) has been detected in oligodendroglia, astroglia and axons, but not in microglia, in acute Type III MS lesions [12]. In our CNS glial cultures, we found predominately downregulation of genes associated with mitochondria. Th1 cytokines upregulated genes for hexokinase II and superoxide dismutase 2 (Mn++ SOD2), and downregulated genes for NADH dehydrogenase (Complex I), COX VIa and COX VIc (Complex IV), ferredoxin 1, and perhaps for 16s ribosomal RNA, which is a component of the large subunit of the mammalian mitochondrial ribosome, responsible for synthesis of 13 proteins localized in the inner mitochondrial membrane [105]. This latter finding has yet to be confirmed. M/M cytokines upregulated genes for SOD2 and down regulated the genes for NADH dehydrogenase and creatine kinases. Th2 cytokines down regulated genes for NADH dehydrogenase and 16S ribosomal RNA. Th1 down regulated the gene for SOD3, an extracellular Cu^++^/Zn^++ ^SOD. None of the cytokine mixtures had an effect on the gene for the mitochondrial protein Cu^++^/Zn^++ ^SOD1; some familial forms of amyotrophic lateral sclerosis (ALS) are associated with mutations in this gene [106]. One could postulate that inflammatory cytokines, perhaps products of activated microglia, at first stimulate transcription of genes for some mitochondrial enzymes, but decreased expression of the 16S subunit of the mitochondrial ribosome could lead to ongoing downregulation of genes and their proteins critical for mitochondrial function in oligodendrocytes and neurons.

### Glutathione-related

We observed effects on the genes for several proteins involved in glutathione metabolism and secretion. Glutathione serves a protective function by reducing the effect of free radicals produced via oxidative stress [107,108], and the cytokine mixtures had significant effects on genes for several proteins involved in synthesis, regulation and release of glutathione. Th1 upregulated the gene for P-glycoprotein/multidrug resistance protein 1/MDR1 (P-glycoprotein/ABCB1), as did M/M cytokines. MDR1, which in addition to inhibiting the therapeutic effects of drugs, has effects on vascular structures and influences secretion of glutathione by cells such as astrocytes [109-111]. In addition to astrocytes, it has been detected in microglia, oligodendrocytes, endothelial cells and neurons [112,113]. Glutathione is more abundant in astrocytes than in other brain cell types, which may contribute to the relative resistance of astrocytes to ischemia and other pathologic processes that involve oxidative stress. Changes in glutathione may also contribute to the relative vulnerability of oligodendrocytes and precursors at different stages of maturation to oxidative stress [107,114-116]. Glutathione also modulates prostaglandin metabolism [117]. We describe cytokine modulation of expression of several genes associated with prostaglandin metabolism [15], and prostglandin D synthase has been reported to be upregulated in MS lesions [2].

### Transcription factors

Some genes for transcription factors were upregulated, quite predictably, such as NF-κB p105 in the presence of IL-1 or TNF-α (Th1 and M/M cytokines). Cyclic AMP response element binding protein 1 (CREB) was upregulated in response to Th1 cytokines.

The upregulation of the gene for CREB by Th1 cytokines may result from the effect of TNF-α[118], although M/M cytokines which also contain this cytokine did not appear to have the same effect.

Jagged 1 is a transcription factor reported to be upregulated in astrocytes by TGF-β and through Hes and notch1 leads to inhibition of myelination [119], although its presence may not be necessary to inhibit myelination [120]. Given the report of upregulation of jagged 1 by TGF-β in astrocytes [119], an unexpected finding in our experiments was upregulation of jagged 1 by Th1 and M/M cytokine mixtures which do not contain TGF-β and the failure of the Th2 cytokine mixture, which contains this cytokine/growth factor, to upregulate jagged 1. These differences could relate to differences in the target tissues (single cell types versus mixtures of different cell types), species and/or effects of a single cytokine versus mixtures of cytokines, effects of some of the induced proteins and their influence on downstream signaling. At 6 hours none of the cytokine mixtures had any discernable effect on expression of notch1 or Hes.

In addition we observed effects on gene expression of other transcription factors including hepatic nuclear factors (HNF), POU and elongation initiation factor 5, important in initial stress responses.

### Nuclear receptors

PPARs are nuclear receptors originally associated with lipid metabolism but subsequently found to also be involved in cellular differentiation. Th1 and M/M cytokines both upregulated the gene for PPAR δ and down regulated the gene for PPAR γ, whereas Th2 cytokines down regulated the gene for PPAR γ and had no effect on the gene for PPAR δ. TNF-α, a component of both the Th1 and M/M cytokine mixtures, has been reported to down regulate the gene for PPAR δ [121]. This is another potential example of differences in the effects of single cytokines versus mixtures of cytokines. Ligation of PPAR γ results in down regulation of inflammatory responses and can inhibit EAE [122,123] and has lead to studies to evaluate such agents, which are used in the treatment of diabetes and hyperlipidemia, as therapy for MS. Activation of PPAR δ with different ligands than those that react with PPAR γ causes activation and accelerates differentiation of oligodendrocytes *in vitro *[124]. How the differential regulation of the PPARs affects inflammation and the potential for favorably influencing remyelination through these receptors is not as yet clear.

### Signaling

Th1 and M/M cytokines markedly upregulated Janus kinase 2 (JAK) as well as several members of the STAT family, in keeping with the well known activation of the JAK/STAT pathway by inflammatory cytokines. Studies in brain ischemia indicate that increased signaling via the JAK/STAT pathway occurs predominantly in microglia rather than astroglia or neurons [125]. We found a 10 fold increase in STAT 1 with Th1 cytokines, consistent with an increase in STAT 1 identified by gene array analysis in both chronic and active MS lesions [126].

The gene for homer, a key protein in Group I metabotropic GluR signaling, was upregulated by Th1 and Th2 cytokines [127-130]. In addition there were effects on expression for genes of protein kinase C, protein kinase A, protein tyrosine kinase 2B, CaM kinase II, Rho family GTPase, receptor serine threonine kinase, lyn protein non-receptor kinase and fyn-related kinase. Fyn, the only src family kinase, is upregulated during oligodendrocyte differentiation [131] and signals through Rho family GTPases to regulate their morphologic maturation [132].

### Cytoplasmic transport and degradation of proteins

Th1 cytokines upregulated the genes for α synuclein and for several proteasome proteins and ubiquitin-like protein (NEDD 6), and down regulated the genes for proteasome subunit R-RING (although as noted upegulated other transcripts of the R-RING subunit), β synuclein (SYN 2), receptor serine threonine kinases, ubiquitin ligase (NEDD 4), and ubiquitin conjugating enzyme E2D 2. M/M cytokines upregulated the genes for NEDD 6 and SYN2, and downregulated the genes for ubiquitin-conjugating enzyme E2D 2, NEDD 4 and proteasome subunit R-RING. Th2 cytokines downregulated the genes for ubiquitin conjugating enzyme E2D 2 and similar to ubiquitin-conjugating enzyme E2D 2. If changes in the expression of these genes result in changes in the level of these proteins, it would imply that inflammation could contribute to the changes in these proteins seen in sporadic forms of several degenerative disorders where synucleins and ubiquitin aggregation have been described [133,134]. The synucleins, considered neuronal proteins, are involved in synaptic function and have chaperone functions as well [135,136]. Th1 cytokines upregulated α-synuclein, which has been detected transiently in rat oligodendrocytes *in vitro *[137] and in inclusions in glial cells in some CNS diseases including multisystem atrophy (MSA) [138]. Proteasomes are involved in transport of protein degradation products as well as in transport of MHC proteins and antigen within antigen presenting cells (APC).

Heat shock proteins (HSP) are upregulated in response to several types of cell stress stimuli [139]. One of several functions of HSP is acting as chaperones to help in normal transport of other proteins within the cytoplasm of many cell types. M/M cytokines upregulated the gene for heat shock protein (HSP) 70 kDa. Interestingly upregulation of the gene for α/β crystalline which also serves as a stress response protein has been reported to be increased in MS lesions [2].

### Lipid synthesis

Th1 cytokines altered gene expression for several enzymes involved in synthesis of fatty acids and phospholipids (Table 2). Both Th1 and M/M cytokines downregulated message of the gene for HMGCoA reductase, the principal regulatory enzyme for cholesterol and other isoprenoids. Interestingly statins, which are inhibitors of this enzyme, are being tested as treatment for MS [139-142] because they inhibit experimental autoimmune encephalomyelitis (EAE), an animal model for MS. The mechanisms include decreased farnesylation causing a Th1 to Th2 shift and monocyte/macrophage inflammation [141,143-145], and perhaps alteration of other signaling pathways [16].

UDP-glucose:ceramide glycosyltransferase is upregulated in the presence of TNF-α (Th1 and M/M cytokines). This enzyme is involved in ceramide metabolism as part of both ceramide induced cell death via TNF-R type I signaling pathways, as well as catalyzing the initial step in ganglioside synthesis. Th1 cytokines also downregulated the gene for UDP-galactose ceramide galactosyltransferase (member 8 of the UDP-glucuronosyl transferase family). This enzyme is markedly upregulated during differentiation of oligodendroglia and synthesizes galactocerebroside, the major glycolipid of myelin and precursor to sulfatide. An early response to inflammatory cytokines has not been previously reported for the gene or the protein.

Notably Th1 cytokines upregulated the gene for phospholipid scramblase, which translocates phospholipids from one surface of the plasma membrane to the other.

In our initial paper we reported that Th1 and M/M cytokines induced robust upregulation of genes for ABC transporter 1, which among its several functions, translocates phosphatidyl choline and cholesterol to the outer membrane leaflet in astrocytes and neurons [146], and for ABC transporter 2, active in oligodendrocytes during myelination [147]. The ABC transporters are also important in intraceullar transport of other proteins including peptide epitopes with MHC class I molecules [15].

### Lipid signaling

Th1 cytokines downregulated the gene for diacyl glycerol kinase beta, which phosphorylates diacyglycerol to produce phosphatidic acid, leading to termination of diacylglyceryol signaling via PKC, Ras GTPase and other signaling pathways.

IL-2, one of the components of the Th1 mixture, upregulates diacylglycerol kinase in myelin [148], raising the possibility that oligodendroglia are upregulating this gene in response to the Th1 cytokines. In addition, the gene for CDP-diacylglycerol synthase, the enzyme that synthesizes phosphatidyl inositol from phosphatidic acid, is robustly upregulated by both Th1 and M/M cytokines, suggesting a switch from diacylglycerol to phosphatidyl inositol mediated signaling pathways. Conversely, the gene for myo-inositol monophosphatase was downregulated by M/M cytokines; this enzyme, the key enzyme inhibited by lithium, generates free inositol from inositol-3-phosphate derived from glucose-6-phosphate, and regulates levels of inositol available for synthesis of phosphatidyl inositol and its multi-phosphorylated derivatives critical for intracellular signaling and trafficking as well as calcium homeostasis. It is of note that lithium is currently being evaluated as a treatment of amyotrophic lateral sclerosis based on its effects on inositol pathways [149-152].

Sphingosine-1-phosphate plays a key role in cell survival and inflammatory responses [153]; the gene for one of its receptors, EDG (endothelial sphingolipid GPCR) was down regulated by both M/M and Th2 cytokines. There has been a phase II trial in patients with MS of an oral agent called FTY72, which binds to the EDG (S1P) receptor (endothelial differentiation sphingolipid G-protein coupled receptor) [154]. A large Phase III study is underway. Inhibition of this receptor both blocks emigration from and favors homing of lymphocytes to secondary lymph structures, ostensibly without affecting T-cell viability or inhibiting memory T-cells. In experimental animals other inflammatory cells, such as monocytes and mature dendritic cells, are also affected and the protein is also found on endothelial cells. The drug has also been used in studies of treatment of other immune disorders [155-160] The S1P receptor EDG is also found in the CNS on glial cells [161-163]. The roles of S1P and its G-coupled receptor in the normal CNS are not known. It has recently been shown that activation of S1P results in changes in glial cells *in vitro *[164,165]. If FTY720 gains access to the CNS there is the potential to modulate the activity of S1P with uncertain consequences for the patient.

### Steroid and vitamin D related

Several genes coding for enzymes involved in steroid metabolism were downregulated by each of the three cytokine mixtures, including the gene for testosterone 6-beta-hydroxylase, markedly downregulated by M/M cytokines. Of note, Th1 cytokines upregulated the gene for vitamin D3 25-hydroxylase, the enzyme catalyzing the first step in activation of dehydrocholesterol to the active hormone, 1, 25-hydroxy vitamin D3. However, both M/M and Th2 cytokines down regulated 25-OH vitamin D3 24-hydroxylase, a key step in the inactivation of the active form of vitamin D3 [166]. Both are mitochondrial enzymes and members of cytochrome p450 family. In several studies vitamin D3 dietary supplementation prevented the onset and progression of EAE. In MBP-induced EAE in mice, the treated animals showed marked decreases in chemokines, iNOS and CD11b+ recruitment into the CNS, perhaps due to activated T cell apoptosis [167]. One large study found that vitamin D3 supplementation reduced the risk of developing MS [168], while four smaller studies suggested a reduction in exacerbations (reviewed in Brown, 2006) [169]. Our findings suggest that both M/M and Th2 cytokines might act to attenuate the effects of the active forms of vitamin D3.

### Miscellaneous proteins

The classically proinflammatory Th1 and M/M cytokines markedly upregulated the gene for iNOS, a critical protein in generation of NO, which gives rise to related reactive oxygen species such as peroxynitrite [170]. Increases in iNOS have been reported in the CNS in EAE and in MS [171-174]. There is evidence that NO could directly or indirectly, by forming peroxynitrite, damage oligodendrocytes, myelin and neurons/axons [175]. Reactive nitrogen species can also influence neuronal Na channels and thus cause damage, especially with rapid firing bare axons [171,176]. It has also been suggested that NO could have an immumodulatory effect on inflammatory cells. NO production in inflammatory cells and in glial cells is induced by iNOS. As described in Results, employing QRT-PCR we confirmed the upregulation of expression of the gene for iNOS by Th1 and M/M cytokine mixtures and also found modest downregulation of the gene in response to Th2 cytokines (Table 3).

Galanin is a peptide in the CNS and PNS which is upregulated in response to injury [177,178]. While originally described in various neurons it has been demonstrated in glia as well.[179,180] and has a positive effect on neurite growth, cell survival and regeneration [181-183] as well as involved in interactions with hormones [184,185], pain signaling pathways [186,187] and other CNS functions [188,189]. Galanin receptors are also co-localized with cholinergic receptors in astroglia [190]. During oligodendrocyte differentiation, the gene for galanin is markedly downregulated [191]. Therefore upregulation by Th1 and M/M cytokines may represent an early attempt of oligodendroglia to return to a less differentiated state, one capable of proliferation.

The alpha-fetoprotein that is increased in the serum of women in the last trimester of pregnancy has been shown to have immunosuppressive effects in EAE as well as in experimental autoimmune myasthenia gravis (EAMG) [192-195]. It also can suppress autoreactivity *in vitro *to two respective autoantigens, MBP and acetylcholine receptor (AChR). This has lead to the suggestion that it may be one of several factors responsible for inhibition of disease activity during the third trimester of pregnancy in patients with MS as well as MG. It is of some interest that expression of the gene for this potentially immunosuppressive protein is downregulated by Th1 and M/M cytokines.

Nestin, an intermediate filament protein, is a marker of early neuronal cell development. It is also a marker of other progenitor cells, particularly glial cells in the CNS, and may be involved in cell proliferation [196-199]. The proinflammatory Th1 cytokine mixture down regulated the gene for nestin, which would be compatible with an inhibitory effect of such cytokines on neuronal and glial cell precursors.

VIPR2 binds VIP, a peptide shown to induce release of cytokines and other factors from glial cells [200,201]. Downregulation of this protein by Th1 cytokines secreted by infiltrating inflammatory cells or endogenous glia would inhibit the release of both cytokines and growth factors by glial cells.

We detected M/M induced upregulation of a gene transcript for angiotensin receptor 2 (ATR2), whereas Th1 cytokines down regulated expression of the same transcript and Th2 cytokines down regulated a different ATR2 transcript. M/M cytokines upregulated the gene for ATR 1. ATR 2 is expressed by endothelial cells as well as glial cells. ATR1 is also expressed by endothelial cells as well as other cells within the CNS. Angiotensin and ATR are involved with interactions with VEGF and other molecules and may be involved in CNS cell death via apoptosis [202-204]. Increased expression of the gene for angiotensin, the ligand for angiotensin receptors, has been described in studies of MS brain tissue [205,206].

Two unexpected and novel findings were the marked decreases in expression of the genes for chapsyn-110 and beta tubulin by Th2 cytokines. Chapsyn-110 is a member of the PSD95/SAP90 protein family. The protein is found in postsynaptic densities in somatic/dendritic neuronal processes, and interacts with the C-terminus of subunits of the NMDA GluR and shaker-type potassium channel [207,208]. The protein is linked indirectly to microtubules and involved in clustering of the receptors and ion channels; its presence and function in glia have not been previously reported. The marked down regulation of chapsyn-110 along with that of beta tubulin in glia may lead to potentially neuroprotective disruption of signaling through NMDA receptors and potassium channels in these cells.

Ceruloplasmin is a metal binding protein which is increased in response to inflammatory signals. In the brain ceruloplasmin is important as a binder of iron, and in the absence of ceruloplasmin (aceruloplasminemia), iron is able to induce tissue injury by increasing lipid peroxidation [209-211]. M/M cytokines upregulated the expression of the gene for ceruloplasmin whereas Th1 and Th2 cytokines had no effect. Effects on genes for iron binding proteins if resulting in sufficient increase in protein would down regulate free iron induced lipid peroxidation, whereas a reduction or even a failure of increase in such proteins could result in cell damage or even death.

Caveolins are a group of proteins that are important in the structure of cell membranes including neurons and myelin. They are components of the so called "lipid rafts", important constituents of plasma membranes. Caveolins 1, 2 and 3 are upregulated in spinal cord of rat with EAE with caveolin 3 being expressed by astrocytes [212], although at 6 hours *in vitro *Th1 cytokines down regulated the expression of the gene for caveolin 3.

Arginase 1 is involved in synthesis of polyamines which have been shown to improve axonal regeneration on myelin substrates [213]. Th2 upregulated the gene for this protein, which would favor axonal regeneration. Th2 cytokines, particularly IL-4, stimulate production of arginase by macrophages, and there is an inverse relationship between production of iNOS induced by Th1 cytokines and arginase induced by Th2 cytokines in these cells [214-216]. By inhibiting production of nitric oxide, arginase may also play a neuroprotective role for motor neurons deprived of trophic factors [217]. Recently, loss of arginase 1 was shown to increase proliferation of neural stem cells [218]. One could postulate that the microglia may be the glial cells upregulating the gene for arginase in our system.

## Overview

In this paper and the preceding two [15,16], we have identified responses to cytokines that would be predicted from analysis of MS tissue, others identified following treatment of individual glial types in culture, and yet others that have not been previously reported. Among the genes predicted from analysis of MS plaques are those related to hypoxic/ischemic responses, inflammatory responses and neuroprotective responses. Most strikingly, our finding that transcription of these genes in glia is changed within 6 hours of exposure to the cytokines implicates the glia as primary responders in the amplification or suppression of damage in white matter. In this paper, we report early changes in a wide variety of genes related to neurotransmitter signaling and ion homeostasis in glial cells. The most striking changes were the decreases induced by Th1 cytokines in dopaminergic receptors, metabotropic glutamate receptor 7b, and a receptor for neuropeptide Y. Identification of which glial type is responding and whether these changes result in long-lasting changes in gene expression, function, and interaction with neurons promise to be informative. With regard to changes in mitochondrial enzymes, the pattern of changes with Th1 cytokines was quite distinct from that seen with M/M cytokines, while Th2 cytokines induced only a few more modest changes. With Th1 cytokines, marked downregulation of the COX VI subunit was seen; this differs from the decrease in the COX IV subunit reported in MS tissue, and may provide a clue to the very earliest changes occurring in mitochondrial function in glia exposed to proinflammatory cytokines, as may the very early downregulation of the 16s mitochondrial ribosomal RNA, which would effect all of the 13 mitochondrial encoded genes. Upregulation by Th1 of genes for transcription factors such as junB, NF-κB and CREB might be predicted, while the decreases in HNF3 and 4 and the increase in the genes for the fox-1 homolog and jagged 1 by Th1 cytokines in glia have not been previously reported. Again, the many changes seen in expression of genes for proteasome, ubiquitin and synuclein proteins with Th1 cytokines might be anticipated, but stand in contrast to the relatively few changes seen in response to M/M and Th2 cytokines. Finally, lipid synthesis and signaling pathways have not been extensively explored in glia in response to cytokines; most notably, decreases by Th1 at 6 hours in the genes coding for synthesis of galactocerebroside implicate changes in oligodenroglial function, since the lipid serves as the precursor for sulfatide, shown to be critical for maintaining normal architecture and function at the nodes. The decrease in the gene for diacyl glycerol kinase and increase in CDP-diglyceride synthase suggests an early switch in signaling pathways within glia.

Table 4 summarizes the largest changes seen with each of the three cytokine mixtures, with the 12 most upregulated genes arranged in order from highest to lowest, and the 12 downregulated genes from most downregulated to least downregulated. While the magnitude of change in gene expression does not necessarily reflect the extent of biological relevance, the summary illustrates a number of changes in common between Th1 and M/M cytokines, as predicted by their predominance of proinflammatory cytokines. Very few genes were upregulated by Th2 cytokines in the categories analyzed in this study, only the 12 genes shown in the table.

**Table 4:** Summary of most upregulated and downregulated gene expression

**MOST UPREGULATED**	**MOST DOWNREGULATED**
**Th1**	**Th1**
nitric oxide synthase 2 (iNOS)	neuropeptide Y receptor 5
STAT 1	dopaminergic receptor A1
CDP-diacylglycerol synthase	dopaminergic receptor D3
proteasome (prosome, macropain) alpha 6	glutamate receptor, metabotropic 7b.
proteasome subunit R-RING	Na+ channel, voltage-gated, 1 alpha
P-glycoprotein, multi-drug resistance 1	K+ channel, voltage-gated, shaker related 2
Janus kinase 2 (JAK 2)	hepatic nuclear factor 4 alpha
fox-1 homolog	PPAR gamma
protein tyrosine kinase 2B	fatty acid CoA ligase, long chain 4
caspase 7	17-beta hydroxy sterol reductase, type 1
cytosolic acyl CoA thioesterase 1	diacyl glycerol kinase beta
superoxide dismutase 2	ubiquitin-conjugating enzyme E2D 2
	
**M/M**	**M/M**
nitric oxide synthase 2 (iNOS)	neuropeptide Y receptor 5
CDP-diacylglycerol synthase	glycine receptor, alpha 1 subunit
superoxide dismutase 2	Na+ channel, voltage-gated, 1 alpha
STAT 5a	testosterone 6-beta hydroxylase
P glycoprotein (multi-drug resistance)	ubiquitin-conjugating enzyme E2D 2
Janus kinase 2 (JAK 2)	EDG, endothelial sphingolipid GPCR
homer	dopaminergic receptor D3
NF kappa B, p105 subunit	glutathione S-transferase, Yc2 subunit
purinergic P2Y receptor 2	beta tubulin 2b
caspase 7	hepatic nuclear factor alpha
protein tyrosine kinase 2b	chapsyn-110
Ca++ ATPase, plasma membrane 1	PPAR gamma
	
**Th2**	**Th2**
CDP-diacylglycerol synthase	chapsyn-110
low density lipoprotein receptor 1	dopaminergic receptor A1
arginase 1	beta tubulin 2b
fox-1 homolog	dopaminergic receptor D3
stress activated protein kinase alpha II	Ca++ channel, voltage-gated, L type alpha 1D
K+ inwardly-rectifying channel, J3 (GIRK1)	preprotachykinin A (substance P precursor)
jun B	serotonergic receptor 4
purinergic P2Y receptor 2	purinergic receptor P2X, ligand-gated 3
glycine transporter 1	angiotensin receptor, type 1b
carbonic anhydrase II	ubiquitin-conjugating enzyme E2D 2
CREB	K+ channel, voltage-gated S1
superoxide dismutase 2	25-OH vitamin D3 24-hydroxylase

### Vascular/Ischemia/Hypoxia

It has been reported that certain MS lesions have features characteristic of ischemic or hypoxic injury to oligodendrocytes [12,219] although inflammatory cells, particularly macrophages, are present in the lesions. Studies of normal appearing white matter in MS, employing gene array technology, have also shown changes in patterns of gene regulation consistent with ischemia and the response to ischemia [9]. It has also been suggested that local angiogenesis occurs in EAE and in MS [220]. We have identified early effects of these cytokine mixtures on molecules that are important in vascular pathology and angiogenesis as well as upregulated in ischemia and hypoxia. Obviously this includes a vast number of genes and gene products involved in transcription, cell signaling, mitochondrial function and apoptosis along with many others.

In addition to the changes in genes for proteins associated with apoptosis and mitochondria, in our current and prior studies [15,16], we found that Th1 cytokines upregulated other genes reported to be regulated in the CNS in ischemia including adhesion molecules (ICAM-1, VCAM), cytokines and chemokines and their receptors (IL-1β, MCP-1), death and survival proteins (Bcl-X), proteases and inhibitors (MMP-9) and growth factors (FGF 1 and NGFRp75). Th1 cytokines did not affect the gene for e-selectin but upregulated the gene for its ligand. Among other genes for proteins regulated in CNS ischemia, Th1 cytokines down regulated genes for neurotrophins and their receptors (BDNF, NT3 and trkB), and cytokines, chemokines and their relevant receptors (several related to TGF-β). M/M cytokines upregulated genes for cell adhesion molecules (ICAM-1, VCAM), HSP 70, cytokines, chemokines and receptors (IL-1β, IL-1R type 1, IL-6, MCP-1), FGF 5 and 10, and MMPs and inhibitors (MMP9, TIMP-1) and downregulated genes of interest for response to ischemia, including TGF-β3, NT3, and FGF2. Th2 cytokines upregulated ischemia related genes for growth factors (BDNF, FGF 10 and 14, FGF-R1), cytokines and chemokines and receptors (IL-6, IL-1R type I and TGF-βR2) and down regulated genes for IL-1R type I, and NT3. Differential expression of many of these genes were reported in the NAWM of some patients with MS [9].

As previously reported [15,16], Th1, M/M and Th2 cytokines had varying effects on the genes for molecules that are involved in altering in the cells of the blood brain barrier including several adhesion molecules and MMPs although our cultures do not contain endothelial cells. Some of these molecules are undoubtedly important in glial cells as well. In a previous study, we detected upregulation of the gene for VEGF [16]. Upregulation of VEGF could contribute to endothelial cell proliferation seen in some MS lesions producing local hypoxia and oligodendroglial death. The function of VEGF in glial cells as well as other non-glial non-neuronal cells, such as pericytes, which conceivably might be in our cultures is not known. Since inflammatory cytokines were able to upregulate the gene for VEGF as well as other genes that are associated with ischemia and the response to ischemia, our data suggests that cytokine release secondary to inflammation can lead to changes compatible with hypoxia and perhaps to induction of hypoxia itself.

We recognize the limitations of microarray analysis as well as gene expression studies since post-transcriptional and post-translational changes are not detected. In addition proteins such as receptors may be present in sufficient amount to be ligated and involved in a biologic process without requiring additional protein in the short run and thus no upregulation of gene for that protein. Nevertheless as a screening technique to obtain an overview of proteins that may be important in a particular process as well as the complexities of the effect of a mixture of factors on a mixture of cells, we believe that this is a promising approach. In addition microarray technology allows discovery of unexpected findings in complex experiments. Such findings may turn out to be both interesting and important.

## Competing interests

RPL has served as a consultant to Teva Neuroscience as well as on the speakers' bureau for Teva Neuroscience. He has also served as a consultant to Genentech, Biogen/Idec, EMD Serono and MediciNova. He has had research funding from Teva Neuroscience, Biogen/Idec, EMD Serono, Bayer Health, Glaxo Smith Kline, BioMS, Abbott, Novartis and Accorda. The other authors declare that they have no competing interests. None of the authors hold stocks or shares in any pharmaceutical company or hold or are applying for any patents relating to the contents of the manuscript.

## Authors' contributions

RPL and JAB were involved in the conception, design, acquisition of data, analysis and interpretation of data, and the drafting of the manuscript. BB and LN performed the tissue culture experiments and BB performed the indirect immunofluorescence experiments. The QRT-PCR was done by DS and for some of the arginase analyses by ER. The gene array procedures were carried out under the supervision of SL and the biometric analysis was carried out by BY. All authors read and approved the final version.
